# Efficacy and safety of esketamine for sedation among patients undergoing gastrointestinal endoscopy: a systematic review and meta-analysis

**DOI:** 10.1186/s12871-023-02167-0

**Published:** 2023-06-13

**Authors:** Xianghong Lian, Yunzhu Lin, Ting Luo, Yang Jing, Hongbo Yuan, Yixin Guo

**Affiliations:** 1 Department of Pharmacy, West China Second University Hospital, Sichuan University, No.20, Third Section, Renmin Nan Lu, Chengdu, Sichuan 610041 People’s Republic of China; 2 Evidence-Based Pharmacy Center, West China Second University Hospital, Sichuan University, Chengdu, People’s Republic of China; 3 Key Laboratory of Birth Defects and Related Diseases of Women and Children, Sichuan University, Ministry of Education, Chengdu, People’s Republic of China

**Keywords:** Esketamine, Gastroscopy, Meta-analysis, Anesthesia, Propofol

## Abstract

**Background:**

Patients who undergo gastrointestinal endoscopy often require propofol-based sedation combined with analgesics. At present, the efficacy and safety of esketamine as an adjunct to propofol for sedation during endoscopic procedures in patients remains controversial. Moreover, there is no universal agreement regarding the appropriate dose of esketamine supplementation. This study aimed to assess the efficacy and safety of esketamine as an adjunct to propofol for sedation during endoscopic procedures in patients.

**Methods:**

Seven electronic databases and three clinical trial registry platforms were searched and the deadline was February 2023. Randomized controlled trials (RCTs) evaluating the efficacy of esketamine for sedation were included by two reviewers. Data from the eligible studies were combined to calculate the pooled risk ratio or standardized mean difference.

**Results:**

Eighteen studies with 1962 esketamine participants were included in the analysis. As an adjunct to propofol, the administration of esketamine reduced the recovery time compared to normal saline (NS). However, there was no significant difference between the opioids group and ketamine group. For propofol dosage, the administration of esketamine required a lower propofol dosage compared to the NS group and opioids group].For complications, the esketamine group had fewer complications compared to the NS group and opioid group in patients, but there were no significant differences between the esketamine group and ketamine group. Notably, the coadministration of esketamine was associated with a higher risk of visual disturbance compared to the NS group. In addition, we used subgroup analysis to investigate whether 0.2–0.5 mg/kg esketamine was effective and tolerable for patients.

**Conclusion:**

Esketamine as an adjunct to propofol, is an appropriate effective alternative for sedation in participants undergoing gastrointestinal endoscopy. However, considering the possibility of its psychotomimetic effects, esketamine should be used with caution.

**Supplementary Information:**

The online version contains supplementary material available at 10.1186/s12871-023-02167-0.

## Introduction

Gastroscopy and colonoscopy are commonly used in the diagnosis of gastrointestinal and colorectal diseases [1, 2]. Current clinical guidelines have recommended the application of anesthesia sedation to relieve the associated physical and emotional stress, which would improve the examination outcomes [3]. In China, the current sedation rate is approximately 50% and has increased rapidly, and the frequently used protocol is the propofol-based sedation combined with other analgesics [4, 5]*.*

Propofol, an ultrashort-acting sedative agent with a shorter recovery time, has been widely used as an intravenous anesthetic in gastrointestinal endoscopy examinations [6–8]. Nevertheless, when propofol was used alone as a sedative for gastrointestinal endoscopy, it had many side effects, such as hypoxemia and major adverse cardiovascular events, which appear to be dose and injection speed related [9–12]. The US Food and Drug Administration recommends that propofol should only be administered by people trained in the administration of general anesthesia [13].Furthermore, propofol as a single drug lacks analgesic effects for painless gastrointestinal endoscopy, and esketamine, midazolam and remifentanil were applied to provide pain relief [14, 15].

Esketamine, a novel N-methyl-D-aspartate receptor antagonist, is the s-enantiomer of ketamine, and its analgesic and sedative effects are twofold higher than those of racemic ketamine [16]. Furthermore, its elimination and recovery time is shorter, and is associated with fewer adverse reactions, such as mental symptoms and respiratory secretions [17]. In addition, its sympathomimetic qualities can counteract the hemodynamic depression of propofol and thus reduce the risk of cardiovascular and respiratory depression during sedation. Therefore, esketamine could be an attractive additive to propofol sedation instead of opioids [18], and some studies have shown that the anesthetic dose of esketamine can produce good sedative and analgesic effects [19, 20].

Considering the previously reported evidence about these complementary effects of esketamine as an adjunct to propofol, the combined use of esketamine and propofol may be a promising approach that could reduce the risk of oversedation of propofol in gastrointestinal endoscopy. However, there is a lack of a high-quality meta-analysis concerning the safety and efficacy of the combined use of esketamine and propofol for gastrointestinal endoscopy. The aim of the study, therefore, was to conduct a systematic review and meta-analysis of randomized controlled trials (RCTs) to investigate the safety and efficacy of esketamine as an adjunct to propofol and the effect of different doses of esketamine for gastrointestinal endoscopy in patients.

## Materials and methods

This meta-analysis was performed according to the recommendations in the Preferred Reporting Items for Systematic Reviews and Meta Analyses (PRISMA) statement and the guidelines described in the Cochrane Handbook [21].

### Search strategy

Our research comprises three English electronic databases (PubMed, Embase, Cochrane Library) and four Chinese electronic databases (China National Knowledge Infrastructure, Wan Fang Database, Chinese Biomedical Literature Database, VIP Database for Chinese Technical Periodicals). Three clinical trial registry platforms were used to find additional studies, including Clinical Trials.gov, the World Health Organization Clinical Trials Registry Platform and Cochrane Central Registry of Controlled Trials. The search strategy was specific for each database and included a combination of the medical subject headings and free text terms for (“esketamine” or “s-ketamine” or “L-ketamine” or “(-)-ketamine”) and (“gastrointestinal endoscopy” or “gastroscopy” or” colonoscopy”). We looked for additional studies in the reference lists of selected articles and contacted the authors if we encountered unclear information. The deadline for all retrieval was February 2023.

### Inclusion/ exclusion criteria

The following criteria were included: (1) intervention: esketamine; (2) comparison: placebo, no intervention or other sedative hypnotics; and (3) type of study: randomized controlled trial (RCT). Exclusion criteria were as follows: (1) patients in intensive care, adult subjects and per protocol use of additional sedative medication other than rescue medication; and (2) studies with incomplete or missing information; and (3) not Chinese or English literature.

The primary outcomes were the following: recovery time (from medication administration to the patients' awakening) and propofol dosage. The secondary outcomes were the following: other adverse events (incidence of nausea and vomiting, injection pain, hypotension, bradycardia, and so on).

### Data extraction

Two authors independently extracted the data based on a previously designed data extraction table. Data extracted were author, year of publication, country, experimental design, sample size, mean age, intervention measure, dose, type of procedure, and any outcome that met the inclusion criteria.

Two independent reviewers screened all the titles and abstracts to determine potential eligible articles. They independently applied the eligibility criteria to perform the final selection. When discrepancies occurred between both reviewers regarding the inclusion of the articles, they discussed and identified the reasons to either include or exclude the articles and then made the final decision. If they could not reach an agreement, the final decision was based on a third reviewer.

### Risk of bias assessment

We used the Cochrane risk of bias tool for RCT studies [22].

### Statistical analysis

Meta-analysis was conducted with RevMan 5.3. The data were pooled and expressed as relative risks (RR) or Mean Difference (MD) with 95% confidence interval (CI). Heterogeneity assessment was formed by I-squared (I^2^) statistics. A fixed effects model was initially conducted. If significant heterogeneity existed among trials (I^2^ > 50%), potential sources of heterogeneity were considered, and where appropriate a random effects model was used [23, 24].

Moreover, subgroup analyses were conducted for all outcomes according to the dosages of esketamine (0.1 mg/kg ~ 1 mg/kg) if applicable.

## Results

### Study search and characteristics

**Fig. 1:**
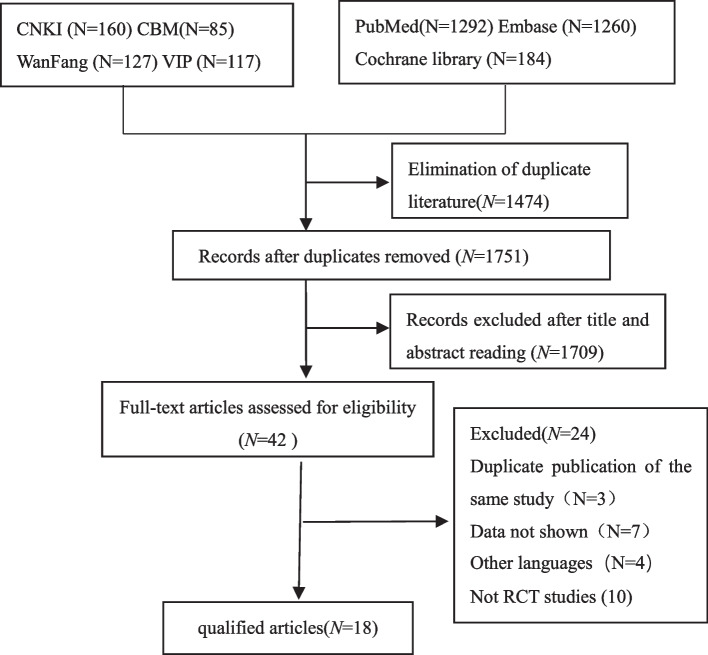
Flow diagram of selecting study

A total of 1660 records were identified for preliminary screening. After screening the titles and abstracts, 18 eligible studies with 1962 participants were included in this meta-analysis (Fig. 1). The dose range of esketamine was 0.1 mg/kg ~ 1 mg/kg (Table 1).

**Table 1:** Characteristics of included randomized-controlled trial

Study ID	Intervention	Sample size	Sex (M/F)	Age (months)	BMI (kg.m^2^)	examination type	ASA	Outcomes
Li P et al. 2022 [25]	0.5 mg/kg esketamine plus 2 mg/kg propofol	114	59/55	47.31 ± 9.30	24.5 ± 3.52	enteroscopy	ASA I ⁓ II	recovery time
	1 ug/kg fentanyl plus 2 mg/kg propofol	114	56/58	46.96 ± 9.44	25.65 ± 2.94			
Kang Y et al. 2021 [26]	0.3 mg/kg esketamine plus 2 mg/kg propofol	30	NA	40–65	19 < BMI < 30	ERCP	ASA I ⁓ II	recovery time
	0.1ug/kg fentanyl plus 2 mg/kg propofol	30	NA					
Shi YH et al. 2020 [27]	0.2 mg/kg esketamine plus 1.5 mg/kg propofol	34	18/16	39.6 ± 2.7	NA	gastrointestinal endoscopy	ASA I ⁓ II	respiratory depression, nausea, low oxygen saturation
	0.5 mg/kg esketamine plus 1.5 mg/kg propofol	34	19/15	37.3 ± 2.1	NA			
	0.5 μg/kg remifentanil plus 1.5 mg/kg propofol	34	17/17	38.2 ± 3.1	NA			
Li CL et al. 2022 [28]	0.3 mg/kg esketamine plus 10 mg/mL propofol (2 ⁓ 4 mg/s)	35	21/14	45.8 ± 11.6	21.2 ± 2.6	gastroscopy	ASA I ⁓ II	propofol dose, recovery time, injection pain, nausea, low oxygen saturation, hypotension, hypertension, bradycardia, tachycardia, dysphoria, muscle tremors
	0.5 mg/kg esketamine plus 10 mg/mL propofol (2 ⁓ 4 mg/s)	35	22/13	43.5 ± 11.5	21.6 ± 2.7			
	NS plus 10 mg/mL propofol (2 ⁓ 4 mg/s)	35	22/13	46.1 ± 12.1	21.6 ± 2.3			
Chen SL et al. 2022 [29]	0.5 mg/kg esketamine plus 2 mg/kg propofol	41	22/19	51.88 ± 6.20	23.67 ± 3.01	gastrointestinal endoscopy	ASA I ⁓ II	recovery time, nausea, respiratory depression, delirium, bradycardia
	NS plus 2 mg/kg propofol	41	24/17	52.36 ± 5.14	23.85 ± 2.34			
Shen K et al. 2022 [30]	0.2 mg/kg esketamine plus 1–2 mg/kg propofol	30	15/15	45.2 ± 9.2	23.9 ± 2.4	gastrointestinal endoscopy	ASA I ⁓ II	propofol dose, recovery time, respiratory depression, body movement, hypotension, bradycardia
	10 ml NS plus 1–2 mg/kg propofol	30	20/10	43.7 ± 13.3	22.3 ± 3.8			
	0.1 mg/kg NS plus 1–2 mg/kg propofol	50	20 /30	49.36 ± 9. 87	23.67 ± 2.73			
Song ZQ et al. 2021 [31]	0.08 mg/kg dezocine plus 1.5 ~ 2.5 mg /kg propofol	40	22/18	71. 3 ± 4. 3	/	gastrointestinal endoscopy	ASA II ⁓ III	respiratory depression, body movement, hypotension
	0.08 μg/kg sufentanil plus 1.5–2.5 mg/kg propofol	40	25/15	72. 8 ± 4. 9	/			
	0.25 mg/kg esketamine plus 1.5 ~ 2.5 mg /kg propofol	40	19/21	68. 8 ± 10. 4	/			
Wan X et al. 2022 [32]	0.25 mg/kg esketamine plus 4–6 mg.kg/h propofol	50	29/21	52.4 ± 11.6	23.9 ± 2.3	gastrointestinal endoscopy	ASA I ⁓ II	propofol dose, recovery time, injection pain, respiratory depression, body movement, dizziness
	NS plus 4–6 mg.kg/h propofol	50	24/26	51.5 ± 1.4	24.3 ± 3.6			
Wang XD et al. 2021 [33]	0.5 µg/kg Dexmedetomidine plus 1 mg/kg propofol	42	30/12	61.6 ± 15.6	23.9 ± 2.3	ERCP	ASA I ⁓ III	recovery time, respiratory depression, nausea, low oxygen saturation, hypotension, bradycardia, dysphoria, tremor
	1 mg/kg esketamine plus 0.4 mg/kg remimazolam	44	NA	60.7 ± 14.8	23.9 ± 2.3			
Xu YF et al. 2022 [20]	0.3 mg/kg esketamine plus propofol	42	16/26	42.3 ± 2.5	NA	gastrointestinal endoscopy	ASA I ⁓ II	propofol dose, recovery time, injection pain, nausea, low oxygen saturation, hypotension, hypertension, bradycardia, tachycardia, dysphoria
	0.05 mg/kg dezocine plus propofol	41	16/25	38.9 ± 2.2	NA			
Yang H et al. 2022 [34]	NS plus propofol	30	10/20	70 [65,88]	24.2 (2.25)	gastrointestinal endoscopy	ASA I ⁓ II	recovery time
	0.25 mg/kg esketamine plus propofol	30	10/20	70 [65, 89]	23.8 (2.73)			
	0.5 mg/kg esketamine plus propofol	30	11/19	69.5 [65,88]	24.8 (2.45)			
Susanne Eberlet al. 2020 [35]	1 μg/kg alfentanil plus propofol	79	39/40	58 [43 to 70]	NA	ERCP	ASA I ⁓ III	propofol dose, recovery time, low oxygen saturation, hypotension, hypertension, bradycardia
	50 μg/kg esketamine plus propofol	83	48/35	63 [52 to 73]	NA			
	0.4 mg/kg remimazolam benzenesulfonate plus 1 mg/kg esketamine	46	31/15	67.62 ± 4.52	NA			
Zheng XS et al. 2022 [36]	0 mg/kg esketamine plus 0.4 mg/kg propofol	23	15/08	8.9 ± 2.6	16.9 ± 2.9	gastrointestinal endoscopy	ASA I –III	propofol dose, recovery time, injection, pain, respiratory depression, nausea, delirium, hypotension, headache, dizziness
	0.25 mg/kg esketamine plus 2.5 mg/kg propofol	23	7/16	9.8 ± 1.9	17.6 ± 2.9			
	0.5 mg/kg esketamine plus 1.5 mg/kg propofol	23	12/11	10.1 ± 3.5	18.2 ± 3.0			
	1 mg/kg esketamine plus 1.5 mg/kg propofol	23	12/11	9.9 ± 2.2	16.5 ± 3.1			
Wang J et al. 2019 [19]	0.5 mg/kg esketamine plus 0.6 mg/kg propofol	16	8/8	32.00 ± 6.19	21.63 ± 1.96	gastroscopy	ASA I–II	recovery time, nausea, delirium, hypertension, tachycardia, muscle tremors, headache, dizziness
	1 mg/kg ketamine plus 0.6 mg/kg propofol	16	8/8	40.00 ± 8.91	23.05 ± 2.69			
Zeng LY et al. 2022 [37]	0.4 mg / kg esketamine plus 1 mg / kg propofol	40	28/12	56 ± 7	23.0 ± 1.2	ERCP	ASA I–II	propofol dose, recovery time, respiratory depression, nausea, dizziness
	5 mg of dizocine plus 1 mg/kg propofol	40	27/13	56 ± 7	23.5 ± 1.5			
Zhan YT et al. 2022 [38]	NS plus 1.5 mg/kg propofol	65	38/27	44.94 ± 10.031	22.67 ± 2.755	gastrointestinal endoscopy(GI)	ASA I–II	propofol dose, recovery time, injection pain, respiratory depression, nausea, delirium, Body dynamic response, Hypoxemia, hypotension, hypertension, dizziness
	0.05 mg/kg esketamine plus 1.5 mg/kg 1.5 mg/kg propofol	65	38/27	42.71 ± 10.148	22.74 ± 2.664			
	0.1 mg/ kg esketamine plus 1.5 mg/kg propofol	65	32/33	45.89 ± 9.292	23.06 ± 2.770			
	0.2 mg/kg esketamine plus 1.5 mg/kg propofol	65	30/35	44.38 ± 10.233	21.99 ± 2.730			
Wang JX et al. 2022 [39]	NS plus 3 mg/kg propofol	30	17/13	9.41 ± 2.06	17.10 ± 2.80	gastro-duodenoscopy	ASA I-II	propofol dose, recovery time, nausea, Hypoxemia, hypotension, hypertension Visual disturbance, dizziness
	0.3 mg/kg esketamine plus 3 mg/kg propofol	30	13/17	9.92 ± 1.87	18.13 ± 3.50			
	0.5 mg/kg esketamine plus 3 mg/kg propofol	30	13/17	8.93 ± 1.95	17.40 ± 3.02			
	0.7 mg/kg esketamine plus 3 mg/kg propofol	30	14/16	9.45 ± 1.66	18.37 ± 3.75			
Feng MM et al. 2022 [40]	NS plus 3 mg/kg propofol	25	14/11	52.6 ± 6.5	22.7 ± 2.2	gastrointestinal endoscopy	ASA I-II	propofol dose, recovery time, respiratory depression, nausea, hypotension, Visual disturbance, dizziness
	0.15 mg/kg esketamine plus 2.5 mg/kg propofol	25	12/13	54.4 ± 8.7	22.8 ± 2.0			
	0.25 mg/kg esketamine plus 2 mg/kg propofol	25	15/10	53.9 ± 6.9	22.8 ± 1.7			
	0.5 mg/kg esketamine plus 1.5 mg/kg propofol	25	11/14	50.7 ± 9.3	23.3 ± 2.3			

### Quality assessment (risk of bias assessment)

**Fig. 2:**
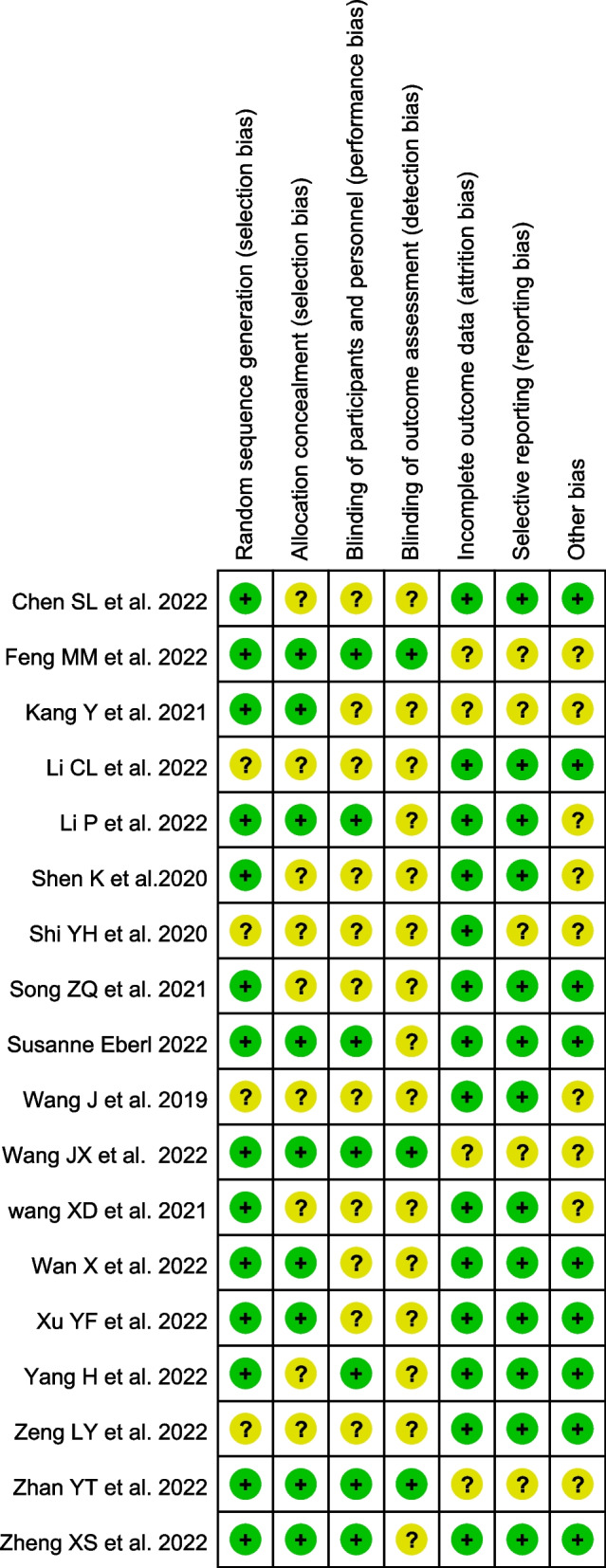
Quality assessment of the included studies. (+ : Low risk of bias –: High risk of bias Yellow grid: Unclear risk of bias)

According to the Cochrane risk of bias tool, 7 aspects were evaluated. In terms of random sequence generation, 77.77% of studies (14/18) with a low risk of bias used an adequate method of random sequence generation, such as using a random number table or a computer-generated random number table. In terms of allocation concealment, 50% of studies (9/18) mentioned allocation concealment. Regarding the blinding of participants and personnel, 38.88% of studies (7/18) performed on the blinding of participants and personnel, such as using computer distribution in the center. For incomplete outcome data, 77.77% of studies (14/18) reported complete outcomes. In terms of selective reporting, 72.22% of studies (13/18) reported no selective reporting with checking protocols. Blinding of outcome assessment and other biases were vague in the majority of trials (Fig. 2).

### Publication bias

We evaluated the publication bias through visual inspection of the funnel plots. No obvious publication bias was found (Supplementary Figure S6).

## Outcomes

### Recovery time

Fifteen studies including a total of 1654 patients provided data on recovery time [18–20, 25, 26, 28–30, 32, 34–39]. Nine studies included 1009 patients in the esketamine group vs. NS group [28–30, 32, 34, 35, 37–39], five studies included 613 patients in the esketamine group vs. opioids group [18, 20, 25, 26, 36], and one study included 32 patients in the esketamine group vs. ketamine group [19].

**Fig. 3:**
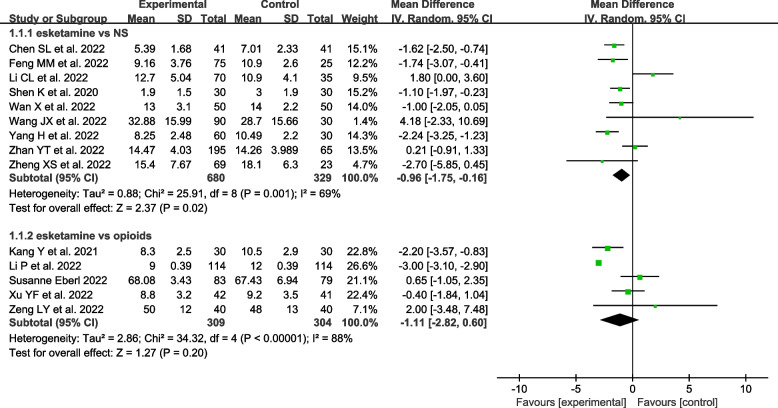
Meta-analysis of the recovery time (min)

Compared to the NS group, the coadministration of esketamine as an adjunct to propofol reduced the recovery time of patients undergoing gastrointestinal endoscopy [MD = -0.96, 95% CI (-1.75, -0.16), I^2^ = 69%, *P* = 0.02]; However, there was no significant difference between the esketamine group and opioids group or ketamine group [MD = -1.11, 95% CI (-2.80, 0.60), I^2^ = 88%, *P* = 0.20] [MD = -4.66, 95%CI (-9.67, 0.35), *P* = 0.07] (Fig. 3). This demonstrated that the coadministration of propofol and esketamine might have a shorter recovery time, which might provide safer and more comfortable sedation in patients during gastroscopy.

We conducted a sensitivity analysis of the primary outcomes by eliminating one included study each time. As a result, removing the study by Li P et al. [25] did change the results (*P* = 0.02, I^2^ = 74%) (Supplementary Figure S4). It was assumed that it originated from the inconsistency in sedation details and different time and sample sources, and no details of these indices were available.

#### Propofol dosage

Seven studies including 820 patients were on propofol [20, 28, 35–39]. Five studies included 657 patients in the esketamine group vs. the NS group [28, 35, 37–39],and two studies included 163 patients in the esketamine group vs. opioid group [20, 36].

**Fig. 4:**
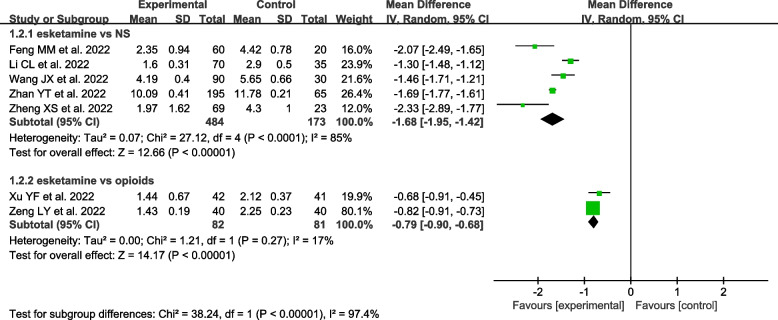
Meta-analysis of the propofol dose (mg/kg)

The results suggested that coadministration of esketamine as an adjunct to propofol required a lower propofol dosage during gastrointestinal endoscopy compared to the NS group and opioid group [MD = -1.68, 95% CI (-1.95, -1.42), I^2^ = 85%, *P* < 0.001)] [MD = -0.79, 95% CI (-0.90, -0.68), I^2^ = 17%, *P* < 0.001] (Fig. 4).

On the other hand, the unit of propofol dosage in these studies [30, 32] was mg (not mg/kg), and the data of these studies cannot be statistically combined into a meta-analysis, which only be described in detail. Shen K et al. [30] and Wan X et al. [32] observed that the esketamine group also significantly reduced the propofol dosage by 171.0 ± 29.2 mg vs.216.6 ± 47.8 mg, and 71.3 ± 5.9 mg vs. 111.8 ± 25.7 mg compared to NS, which was also consistent with the meta-analysis of the propofol dosage above (mg/kg).

### Adverse events

Fifteen studies including 1726 patients reported adverse events [18–20, 25–32, 35–39]. Eight studies included 919 patients in the esketamine group vs. NS group [28–32, 35, 37–39], six studies included 775 patients in theesketamine group vs. opioid group [18, 25–27, 31, 36], and one study included 32 patients in the esketamine group vs. ketamine group [19]. Moreover, the subgroup results of the RR and 95% CI of all complications for esketamine group during gastrointestinal endoscopy in patients were shown in Table 2.

**Table 2:** RR and 95% CI of complications during gastrointestinal endoscopy

**Subgroup** **Complications**	**Control**	**Number of studies**	**Results of heterogeneity test**		**Meta analysis results**	
			***P* value**	**I**^**2**^	**MD (95%** CI**)**	***P* value**
**(A)** injection pain	NS group	4	0.12	48%	0.20(0.08,0.49)	0.0004
**(B)** body movement	NS group	4	0.15	44%	0.76(0.65,0.90)	0.001
	opioids group	2	0.64	0%	-0.11(0.21, -0.00)	0.05
**(C)** hypotension	NS group	6	0.006	69%	0.31(0.22,0.43)	0.003
	opioids group	3	0.47	0%	-0.14(-0.21, -0.06)	0.0002
**(D)** respiratory depression	NS group	6	0.29	19%	0.33(0.19,0.58)	0.0001
	opioids group	4	< 0.001	86%	-0.15(-0.29, -0.00)	0.05
**(E)**nausea or vomiting	NS group	5	0.40	1%	0.78(0.30,2.04)	0.61
	opioids group	4	< 0.001	90%	-0.18(-0.41,0.04)	0.11
**(F)**Hypoxemia	NS group	3	1.05	47%	1.05(0.68,1.62)	0.84
	opioids group	2	0.33	0%	-0.00(-0.06,0.06)	0.94
**(G)** hypertension	opioids group	3	0.07	62%	-0.04(-0.13,0.05)	0.42
**(H)**bradycardia	NS group	3	0.54	0%	0.71(0.14,3.66)	0.68
	opioids group	3	0.06	64%	-0.01(-0.10,0.08)	0.85
**(I)**tachycardia	opioids group	2	0.50	0%	0.03(-0.04,0.10)	0.40
**(J)**delirium	NS group	2	0.11	61%	3.29(0.61,17.83)	0.17
**(K)**dizziness	NS group	6	0.99	0%	1.38(0.94,2.02)	0.10
**(L)** Visual disturbance	NS group	3	0.56	0%	5.84(1.88,18.20)	0.002

**Fig. 5:**
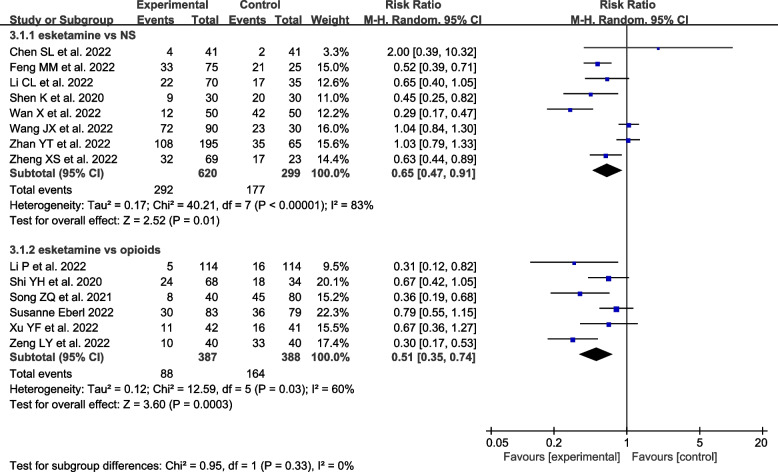
The overall number of complications of esketamine

The results suggested that coadministration of esketamine as an adjunct to propofol had fewer complications in patients compared to the NS group and opioid group [RR = 0.65, 95% CI (0.47,0.91), I^2^ = 83%, *P* = 0.01] [RR = 0.51, 95% CI (0.35, 0.74), I^2^ = 60%, *P* < 0.05] (Fig. 5), but there were no significant differences between the esketamine group and ketamine group [RR = 0.86, 95% CI (0.61,1.20), *P* = 0.37]. Sensitivity analysis for each comparison revealed no robust changes in significance (Supplementary Figure S5).Moreover, subgroup analysis of studies in which esketamine was coadmnistered was shown as follows.

**Fig. 6:**
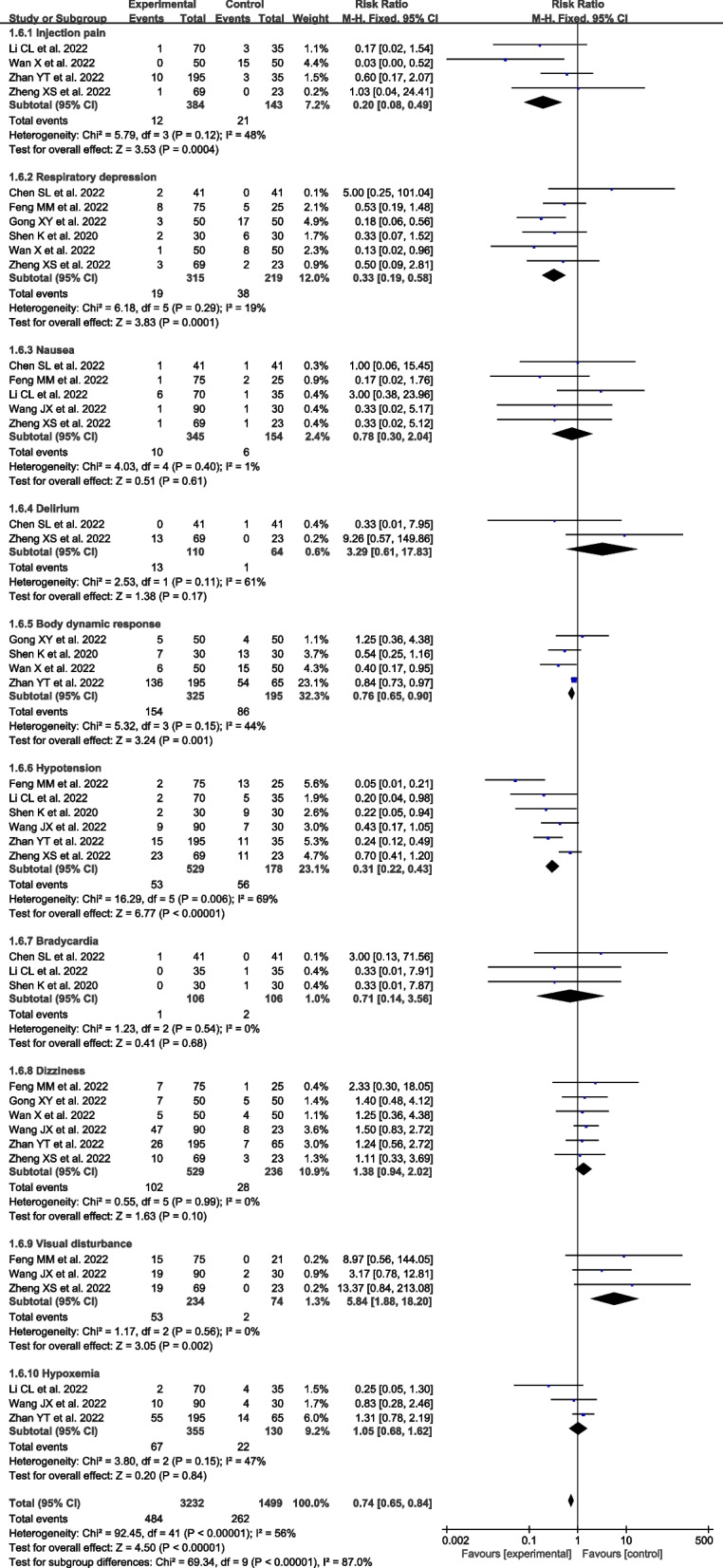
Forest plots of the complications between the esketamine group and the NS group

**Fig. 7:**
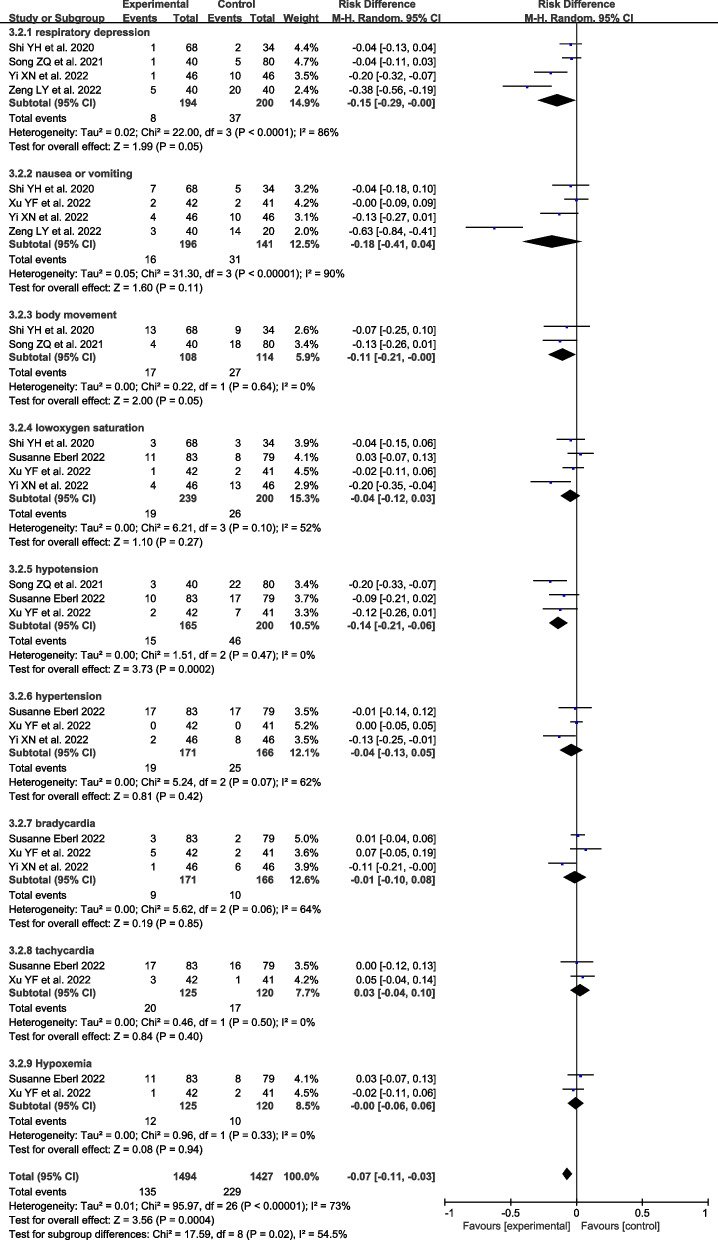
Forest plots of the complications between the esketamine group and the opioids group

Compared to NS group, the coadministration of esketamine resulted in the reduction in injection pain [RR = 0.20, 95% CI (0.08, 0.49), I^2^ = 48%, *P* = 0.0004], body movement [RR = 0.76, 95% CI (0.65, 0.90), I^2^ = 44%, *P* = 0.001], hypotension [RR = 0.31, 95% CI (0.22,0.43), I^2^ = 69%, *P* < 0.00001], respiratory depression [RR = 0.33, 95% CI (0.19,0.58), I^2^ = 19%, *P* = 0.0001], but had no remarkable effect on nausea or vomiting [RR = 0.78, 95% CI (0.30,2.04), I^2^ = 1%, *P* = 0.61], bradycardia [RR = 0.71, 95% CI (0.14,3.56), I^2^ = 0%, *P* = 0.68], delirium [RR = 3.29, 95% CI (0.61,17.83), I^2^ = 61%, *P* = 0.17], dizziness [RR = 1.38, 95% CI (0.94,2.02), I^2^ = 0%, *P* = 0.10], hypoxemia [RR = 1.05, 95% CI (0.68,1.62), I^2^ = 47%, *P* = 0.84]. Notably, the coadministration of esketamine was associated with a higher risk of visual disturbance [RR = 5.84,95% CI (1.88, 18.20), I^2^ = 0%, *P* = 0.002] compared to the NS group (Fig. 6). Compared to opioid group, the administration of esketamine had a fewer risk of hypotension [RD = -0.14, 95% CI(-0.21,-0.06), I^2^ = 0%, *P* = 0.0002], respiratory depression [RD = -0.15, 95% CI (-0.29,-0.00), I^2^ = 86%, *P* < 0.001], but had no remarkable effect on body movement [RD = 0.11, 95% CI (0.21, -0.00), I^2^ = 0%, P = 0.05], nausea or vomiting [RD = -0.18, 95% CI (-0.41,0.04), I^2^ = 90%, P = 0.11], hypoxemia [RD = -0.00, 95% CI (-0.06,0.06), I^2^ = 0%, *P* = 0.94], hypertension [RD = -0.04, 95% CI (-0.13,0.05), *P* = 0.42], bradycardia [RD = -0.01, 95% CI (-0.10,0.08), I^2^ = 64%, *P* = 0.85], tachycardia [RD = 0.03, 95% CI (-0.04,0.10), *P* = 0.40] (Fig. 7).

### Esketamine dosage subgroup analysis results

In addition, we used subgroup analysis to investigate the differential effects of the esketamine dose 0.1–0.15 mg/kg, 0.2–0.3 mg/kg, 0.4–0.5 mg/kg and 0.7–1 mg/kg) on the outcome assessment. The results of the meta-analysis are summarized in Table 3.

**Table 3:** Subgroup analysis results of esketamine dosage

**Subgroup** **Outcomes**	**Control**	**Number of studies**	**Results of heterogeneity test**		**Meta analysis results**	
			***P* value**	**I**^**2**^	**MD or RR (95%** CI**)**	***P* value**
**(A) Recovery time**						
0.1–0.15 mg/kg esketamine	NS group	2	0.0003	92%	-1.76 (-5.13,1.62)	0.31
0.2–0.3 mg/kg esketamine	NS group	8	0.004	67%	-1.03(-1.98,-0.08)	0.03
	opioids group	2	0.08	68%	-1.35(-2.34,-0.35)	0.008
0.4–0.5 mg/kg esketamine	NS group	6	< 0.001	88%	0.24(-1.87,2.35)	0.82
	opioids group	3	< 0.001	90%	-0.93(-4.15,2.29)	0.57
0.7–1 mg/kg esketamine	NS group	2	0.008	86%	-0.12(-12.67,12.42)	0.98
**(B) Propofol dosage**						
0.1–0.15 mg/kg esketamine	NS group	2	0.09	66%	-1.31 (-1.64, -0.99)	< 0.001
0.2–0.3 mg/kg esketamine	NS group	5	< 0.001	89%	-1.50(-1.85, -1.16)	< 0.001
	opioids group	2	0.27	17%	-0.79(-0.90, -0.68)	< 0.001
0.4–0.5 mg/kg esketamine	NS group	4	0.003	78%	-2.87(-3.69, -2.05)	< 0.001
	opioids group	2	< 0.001	99%	-1.47(-2.75, -0.18)	0.03
0.7–1 mg/kg esketamine	NS group	2	0.44	0%	-3.21(-3.80, -2.62)	< 0.001
**(C) Adverse events**						
0.1–0.15 mg/kg esketamine	NS group	2	0.005	87%	0.74 (0.33, 1.66)	0.47
0.2–0.3 mg/kg esketamine	NS group	7	< 0.001	83%	0.58(0.39, 0.85)	0.006
	opioids group	3	0.38	0%	0.49(-0.34,0.72)	0.0002
0.4–0.5 mg/kg esketamine	NS group	5	0.36	8%	0.75(0.60, 0.94)	0.01
	opioids group	4	< 0.001	86%	0.67(0.56, 0.81)	< 0.001
0.7–1 mg/kg esketamine	NS group	2	0.84	0%	1.32 (1.12, 1.54)	0.0007

For recovery time, the coadministration of esketamine resulted in a reduction in recovery time in the 0.2–0.3 mg/kg esketamine groups [MD = -1.03, 95% CI (-1.98,-0.08), I^2^ = 67%, *P* = 0.03][MD = 0.24, 95% CI (-1.87,2.35), I^2^ = 68%, *P* = 0.008]. However, subgroup analysis showed that the recovery time was not significantly different in the 0.1–0.15 mg/kg, 0.4–0.5 mg/kg, and 0.7–1 mg/kg esketamine groups [MD = -1.76, 95% CI (-5.13,1.62), I^2^ = 92%, *P* = 0.31][MD = 0.24, 95%CI (-1.87,2.35), I^2^ = 88%, *P* = 0.82][MD = -0.93, 95% CI (-4.15,2.29), I^2^ = 90%, *P* = 0.57][MD = -4.66, 95% CI (-9.67,0.35), *P* = 0.07)][MD = -0.12, 95% CI (-12.67,12.42), I^2^ = 86%, *P* = 0.98]. For propofol dosage, there was a significant difference between the control group and esketamine group (0.1–0.15 mg/kg, 0.2–0.3 mg/kg, 0.4–0.5 mg/kg and 0.7–1 mg/kg) regardless of the dosage (Table 3). For adverse events, the administration of esketamine had a lower risk of complications at 0.2–0.3 mg/kg and 0.4–0.5 mg/kg esketamine compared to the control [RR = 0.58, 95% CI (0.39, 0.85), I^2^ = 83%, *P* = 0.006][RR = 0.49, 95% CI (-0.34,0.72), I^2^ = 0%, *P* = 0.0002][RR = 0.75, 95% CI (0.60, 0.94), I^2^ = 8%,*P* = 0.01][RR = 0.67, 95% CI (0.56, 0.81), I^2^ = 86%,*P* < 0.001], while there was no significant difference if supplementation was from 0.1–0.15 mg/kg eskatmine [RR = 0.74, 95% CI (0.33, 1.66), I^2^ = 87%,*P* = 0.47]. Notably, the coadministration of 0.7–1 mg/kg esketamine was associated with a higher risk of complications [RR = 1.32,95% CI (1.12, 1.54), I^2^ = 0%, *P* = 0.0007] compared to the NS group (Table 3, Figure S1, S2, S3).

## Discussion

In the present meta-analysis of randomized controlled trials of patients undergoing gastrointestinal endoscopy, esketamine as an adjunct to propofol resulted in a reduction in propofol dosage, recovery time and adverse events compared to the NS group. Furthermore, subgroup analysis showed that 0.2–0.5 mg/kg esketamine was effective and tolerable for patients, which indicated that esketamine is an appropriate effective alternative for sedation with propofol in participants undergoing gastrointestinal endoscopy. However, considering the possibility of visual disturbances, esketamine should be used with caution.

Sedation strategies for gastrointestinal endoscopy have developed rapidly in recent years. Propofol is widely used for intravenous anesthesia, and has the characteristics of depressant effects on the laryngeal reflexes, as well as faster awakening, but it can lead to marked depression of respiratory and angiocarpy parameters [40–43]. Therefore, minimizing these risks is an primary goal to make anesthesia sedation procedures safer. A possible approach was to reduce the propofol dosage using a combination with other substances.

Esketamine is a noncompetitive, N-methyl-D-aspartate receptor antagonist. Recently, esketamine has received wide attention for its potential implications in treatment-resistant depression. In addition to its antidepressant effect, esketamine could also be an effective anesthetic and analgesic agent used for surgical anesthesia [44, 45]. It has analgesic and sympathomimetic properties and is known to cause less cardiorespiratory depression [16, 17]. In addition, Eberl et al, [18] reported that low-dose esketamine reduces the total amount of propofol necessary for sedation during ERCP while providing satisfactory sedative effects. Furthermore, many studies report that a combination of propofol with esketamine may result in a better quality of sedation and analgesia, with shorter recovery time, better satisfaction of patients and fewer respiratory or cardiovascular side effects [46]. Therefore, esketamine could be attractive additive propofol instead of opioids, which may be a promising approach that could reduce the risk of oversedation of propofol in gastrointestinal endoscopy. Recently, Hengrui Medicine Co, Ltd. completed the preclinical study of esketamine and obtained the clinical research approval from SFDA [19]. Thus,it is valuable and urgent to explore the efficacy and safety of esketamine for sedation in gastrointestinal endoscopy. However, no meta study has reported the effectiveness and safety of esketamine adjunct to propofol for sedation during endoscopic procedures in patients.

Recovery time is widely considered by anesthesiologists and endoscopists [47]. Our meta-analysis demonstrated that the coadministration of propofol and esketamine might have a shorter recovery time, which might provide safer and more comfortable sedation in patients during gastroscopy [48].

The dosage of propofol was an important index to evaluate the safety of gastrointestinal endoscopy [49]. Our meta-analysis demonstrated that there were significant differences between the esketamine and control group, which showed that an adequate level of sedation and analgesia could be achieved with less propofol and fewer cardiopulmonary adverse effects.

Furthermore, the study evaluated overall adverse effects among groups. The results suggested that coadministration of esketamine and propofol had fewer complications in patients undergoing gastrointestinal endoscopy. However, there was no significant difference between esketamine and ketamine. Moreover, subgroup analysis of studies showed that the esketamine group had a lower risk of respiratory depression, and hypotension than the NS or opioid group (Table 2), which may be due to the lower doses of propofol and esketamine counteracting hypotension due to its sympathomimetic properties or stimulating breathing by increasing carbon dioxide sensitive ventilation [50, 51]. No significant difference was found in the risk of bradycardia events. In addition, a potential problem of esketamine could be its psychotomimetic effects, such as visual disturbances, and dizziness, which could compromise patient satisfaction. Our meta-analysis also demonstrated that the coadministration of esketamine was associated with a higher risk of visual disturbance compared to the NS group (Fig. 7). However, no significant difference was found in the risk of dizziness events (Table 2), which was probably related to propofol used in clinically relevant dosages suppressing these effects via the activation of GABA receptors [52]. In addition, this is also possible due to only a few studies with limited significance investigating the eventual psychotomimetic effects, such as visual disturbances, and dizziness, that could compromise patient satisfaction.

Furthermore, it is important to note that subgroup analysis is supportive of the main research question. Subgroup analysis for various dosages of esketamine (0.1–0.15 mg/kg, 0.2–0.3 mg/kg, 0.4–0.5 mg/kg and 0.7–1 mg/kg) is needed. For recovery time, there was a significant difference between the 0.2–0.3 mg/kg esketamine groups and the control group. For propofol dosage, significant differences were also observed on 0.1–0.15 mg/kg, 0.2–0.3 mg/kg, 0.4–0.5 mg/kg and 0.7–1 mg/kg esketamine. However, for adverse events, we found that 0.7–1 mg/kg esketamine supplementation was associated with a higher risk of complications among groups, while there was a lower risk of complications compared to the control if supplementation was from 0.2–0.3 mg/kgor 0.4–0.5 mg/kg esketamine. Although higher doses of esketamine have the advantage of reducing propofol consumption, they do not reduce recovery time or adverse reactions (Table 3, Figure S1, S2, S3). Through analysis of the included studies and comprehensive consideration of effectiveness and safety, we deduced that a dose of 0.2–0.5 mg/kg is safe and effective. The use of high doses of esketamine may not be appropriate for OPD procedures and specific patient groups based on the evidence. In contrast, it is important to be aware of the adverse effects of high doses of esketamine in the clinic. Since there were not enough studies in the former analysis, we also tried to use 0.5 mg/kg esketamine as a cutoff value to perform the subgroup analysis, and the results showed the same results as above.

In addition, quality assessment of the studies included in the present meta-analysis was performed. Heterogeneity was identified in the outcomes of recovery time **(**I^2^** = **88%) and propofol dosage (I^2^ = 94%). For propofol dosage, subgroup analysis of studies that used 0.2–5 mg/kg esketamine compared to NS did not change the results but had low heterogeneity (I^2^ = 29%), which suggested that the different dosages of esketamine were one of the reasons for the high heterogeneity. For recovery time, removing the study by Li CL et al. [28] and Li P et al. [25] decreased the heterogeneity of recovery time (*P* < 0.00001, I^2^ = 59%), but did not change the results. It was assumed that the high heterogeneity originated from the inconsistency in sedation details and different time and sample sources, and no details of these indices were available.

## Limitations

Several limitations of the study should be acknowledged. First, due to the limited number of original studies, many results could not be combined. Second, outcome measurements were quite different across individual studies. Therefore, there were only a few RCTs to be statistically analyzed. Recovery time, esketamine supplementation dosage, and different control groups, may have diluted the significance of certain specific results. Furthermore, among the included trials, most of the included studies were conducted in China, which might cause bias. A potential problem of esketamine could be its psychotomimetic effects, such as visual disturbances, vertigo, or nausea, which could compromise patient satisfaction. The main finding of equivocal effect between esketamine and ketamine groups and its visual disturbance and other dissociative symptoms are under-estimated. The small sample size of this study may lead to an underestimation of the adverse effect. Therefore, well-conducted RCTs are urgently needed to evaluate the safety of the combined use of propofol and esketamine on psychotomimetic effects and cognitive impairment after recovery, such as mood and clustered psychological effects and concentration capacity. In addition, the comparison of esketamine with saline is a relatively low standard for the design of high quality RCTs, and there is no registration of this meta-analysis, so this may bias the findings.

## Supplementary Information

**Additional file 1: Figure S1.** Forest plots of the recovery time with different dosage of esketaime (mg/kg).

**Additional file 2: Figure S2.** Forest plots of the propofol dose with different dosage of esketaime (mg/kg).

**Additional file 3: Figure S3.** Forest plots of the complications with different dosage of esketaime (mg/kg).

**Additional file 4: Figure S4.** Forest plots of sensitivity of recovery time.

**Additional file 5: Figure S5.** Forest plots of sensitivity of the adverse events.

**Additional file 6: Figure S6.** The funnel plot of adverse events.

**Additional file 7.**

**Additional file 8: Table S1.** Subgroup analysis results of 0.5 mg/kg esketamine as the cutoff point.

## Abbreviations

RCTs: Randomized controlled trials
NS: Normal saline
MD: Mean Difference
CI: Confidence interval
RR: Risk ratio
HR: Heart rate
PRISMA: Preferred reporting items for systematic reviews and meta-analyses statement
