# Supplementary material for: Efficacy and safety of esketamine for sedation among patients undergoing gastrointestinal endoscopy: a systematic review and meta-analysis
Source: BMC Anesthesiol. 2023 Jun 13;23:204. doi: 10.1186/s12871-023-02167-0 (PMC10262466; doi:10.1186/s12871-023-02167-0)

(A)0.1-0.15mg/kg esketamine

Study or Subgroup	Experimental Mean	Experimental SD	Experimental Total	Control Mean	Control SD	Control Total	Weight	Mean Difference IV, Random, 95% CI
**1.1.1 esketamine vs NS**								
Feng MM et al. 2022	7.4	2.6	25	10.9	2.6	25	49.3%	-3.50 [-4.94, -2.06]
Gong XY et al. 2022	4.62	0.45	50	4.58	0.45	50	0.0%	0.04 [-0.14, 0.22]
Zhan YT et al. 2022	14.2	4.1	130	14.26	3.989	65	50.7%	-0.06 [-1.26, 1.14]
**Subtotal (95% CI)**			**155**			**90**	**100.0%**	**-1.76 [-5.13, 1.62]**

Heterogeneity: Tau² = 5.46; Chi² = 12.93, df = 1 (P = 0.0003); I² = 92%
Test for overall effect: Z = 1.02 (P = 0.31)

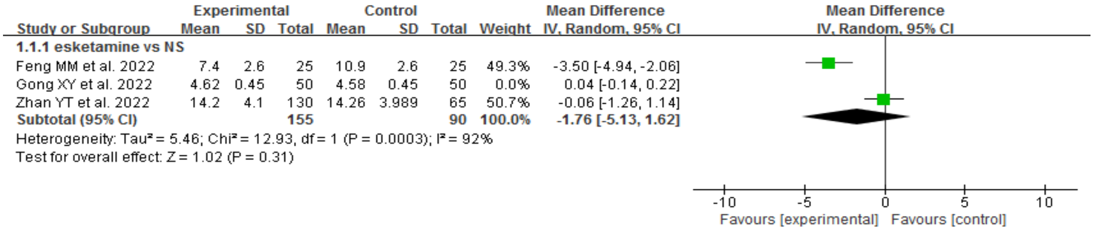

(B)0.2-0.3mg/kg esketamine

Study or Subgroup	Experimental Mean	Experimental SD	Experimental Total	Control Mean	Control SD	Control Total	Weight	Mean Difference IV, Random, 95% CI
**1.1.1 esketamine vs NS**								
Chun-Lin Li 2022	11.1	4	35	10.9	4.1	35	11.7%	0.20 [-1.70, 2.10]
Feng MM et al. 2022	7.4	2.8	25	10.9	2.6	25	14.2%	-3.50 [-5.00, -2.00]
Hua Yang 2022	8.82	2.8	30	10.49	2.2	30	15.7%	-1.67 [-2.94, -0.40]
Kui Shen 2020	1.9	1.5	30	3	1.9	30	18.5%	-1.10 [-1.97, -0.23]
Wang JX et al. 2022	29.45	11.4	30	28.7	15.66	30	1.7%	0.75 [-6.18, 7.68]
Xiao-Su Zheng 2022	16.1	5.4	23	18.1	6.3	23	5.8%	-2.00 [-5.39, 1.39]
Xing Wan 2022	13.8	3.1	50	14.4	2.2	50	17.2%	-0.60 [-1.65, 0.45]
Zhan YT et al. 2022	15.02	3.851	65	14.26	3.989	65	15.2%	0.76 [-0.59, 2.11]
**Subtotal (95% CI)**			**288**			**288**	**100.0%**	**-1.03 [-1.98, -0.08]**
Heterogeneity: Tau² = 1.08; Chi² = 21.03, df = 7 (P = 0.004); I² = 67%								
Test for overall effect: Z = 2.12 (P = 0.03)								
**1.1.2 esketamine vs opioids**								
Ya-Fei Xu 2022	8.8	3.2	42	9.2	3.5	41	49.2%	-0.40 [-1.84, 1.04]
Yu Kang 2021	8.3	2.5	30	10.5	2.9	30	50.8%	-2.20 [-3.57, -0.83]
**Subtotal (95% CI)**			**72**			**71**	**100.0%**	**-1.31 [-3.08, 0.45]**
Heterogeneity: Tau² = 1.10; Chi² = 3.14, df = 1 (P = 0.08); I² = 68%								
Test for overall effect: Z = 1.46 (P = 0.14)								

Test for subgroup differences: Chi² = 0.08, df = 1 (P = 0.78), I² = 0%

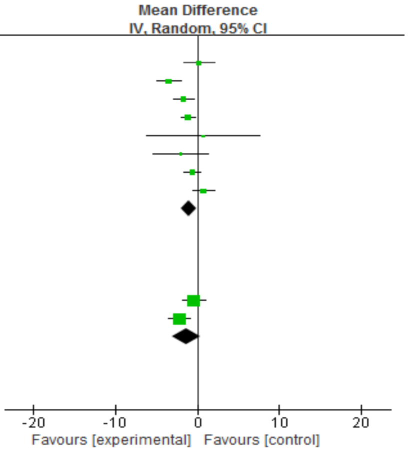

(C)0.4-0.5mg/kg esketamine

Study or Subgroup	Experimental Mean	Experimental SD	Experimental Total	Control Mean	Control SD	Control Total	Weight	Mean Difference IV, Random, 95% CI
**1.1.1 esketamine vs NS**								
Chun-Lin Li 2022	14.3	5.5	35	10.9	4.1	35	18.3%	3.40 [1.13, 5.67]
Feng MM et al. 2022	12.7	3.1	25	10.9	2.6	25	20.5%	1.80 [0.21, 3.39]
Hua Yang 2022	7.68	1.99	30	10.49	2.2	30	21.9%	-2.81 [-3.87, -1.75]
Sheng-Lan Chen 2022	5.39	1.68	41	7.01	2.33	41	22.3%	-1.62 [-2.50, -0.74]
Wang JX et al. 2022	33.5	16.47	30	28.7	15.66	30	5.2%	4.80 [-3.33, 12.93]
Xiao-Su Zheng 2022	17.9	9.9	23	18.1	6.3	69	11.8%	-0.20 [-4.51, 4.11]
**Subtotal (95% CI)**			**184**			**230**	**100.0%**	**0.24 [-1.87, 2.35]**
Heterogeneity: Tau² = 4.97; Chi² = 41.40, df = 5 (P < 0.00001); I² = 88%								
Test for overall effect: Z = 0.22 (P = 0.82)								
**1.1.2 esketamine vs opioids**								
Lingyuan Zeng 2022	56	16	40	55	14	40	15.6%	1.00 [-5.59, 7.59]
Ping Li 2022	9	0.393	114	12	0.393	114	44.7%	-3.00 [-3.10, -2.90]
Susanne Eberl 2022	68.08	3.43	83	67.43	6.94	79	39.8%	0.65 [-1.05, 2.35]
**Subtotal (95% CI)**			**237**			**233**	**100.0%**	**-0.93 [-4.15, 2.29]**
Heterogeneity: Tau² = 6.04; Chi² = 19.07, df = 2 (P < 0.0001); I² = 90%								
Test for overall effect: Z = 0.56 (P = 0.57)								

Test for subgroup differences: Chi² = 0.35, df = 1 (P = 0.55), I² = 0%

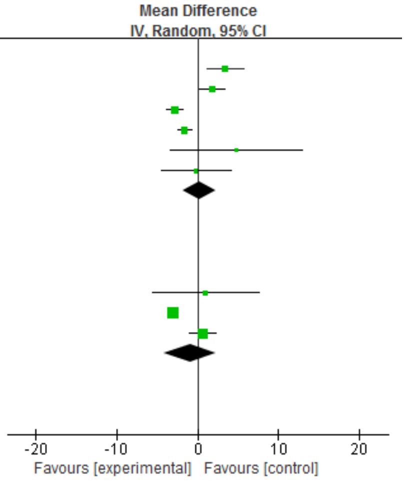

(D)0.7-1mg/kg esketamine

Study or Subgroup	Experimental Mean	Experimental SD	Experimental Total	Control Mean	Control SD	Control Total	Weight	Mean Difference IV, Random, 95% CI
**1.1.1 esketamine vs NS**								
Wang JX et al. 2022	35.67	19.04	30	28.7	15.66	30	44.9%	6.97 [-1.85, 15.79]
Xiao-Su Zheng 2022	12.2	6.1	23	18.1	6.3	23	55.1%	-5.90 [-9.48, -2.32]
**Subtotal (95% CI)**			**53**			**53**	**100.0%**	**-0.12 [-12.67, 12.42]**
Heterogeneity: Tau² = 71.02; Chi² = 7.02, df = 1 (P = 0.008); I² = 86%								
Test for overall effect: Z = 0.02 (P = 0.98)								
**Total (95% CI)**			**53**			**53**	**100.0%**	**-0.12 [-12.67, 12.42]**

Heterogeneity: Tau² = 71.02; Chi² = 7.02, df = 1 (P = 0.008); I² = 86%
Test for overall effect: Z = 0.02 (P = 0.98)
Test for subgroup differences: Not applicable

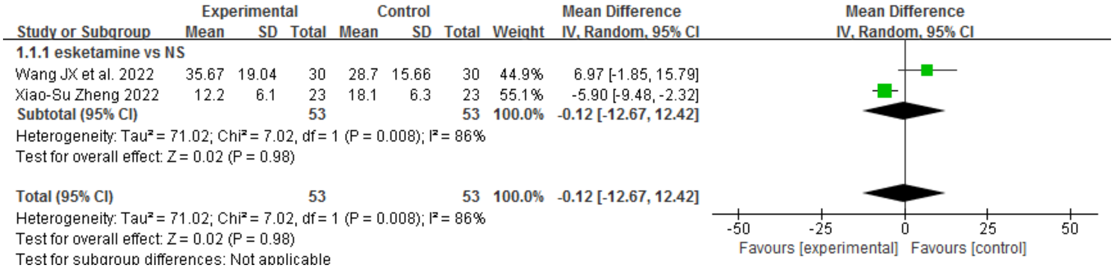

Supplement: Supplementary file 1 — Additional file 1: Figure S1. Forest plots of the recovery time with different dosage of esketaime (mg/kg). [file 12871_2023_2167_MOESM1_ESM.pdf]
