# Supplementary figures and images for: Efficacy and safety of esketamine for sedation among patients undergoing gastrointestinal endoscopy: a systematic review and meta-analysis
Source: BMC Anesthesiol. 2023 Jun 13;23:204. doi: 10.1186/s12871-023-02167-0 (PMC10262466; doi:10.1186/s12871-023-02167-0)

### (A) 0.1-0.15mg/kg esketamine

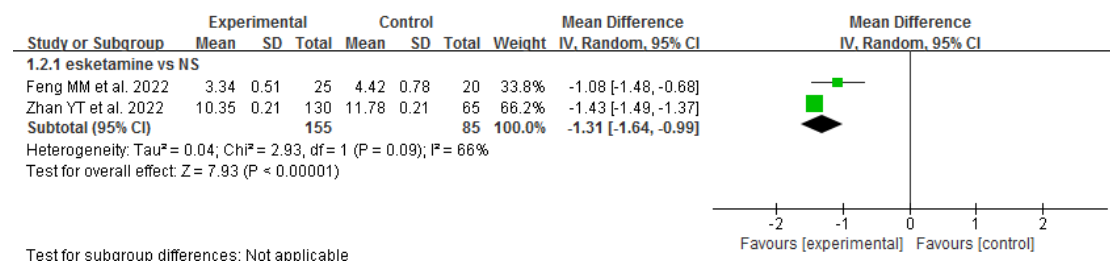

### (A) 0.2-0.3mg/kg esketamine

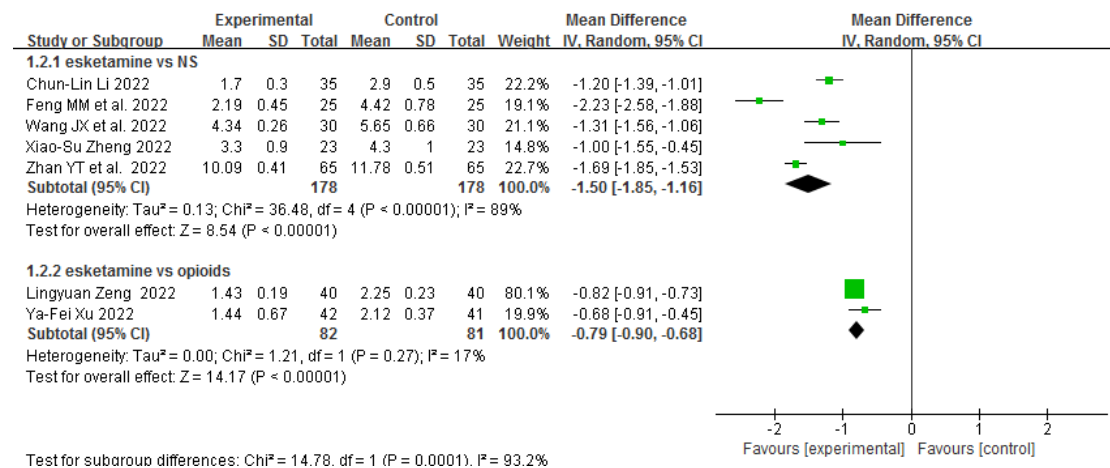

### (B) 0.4-0.5 mg/kg esketamine

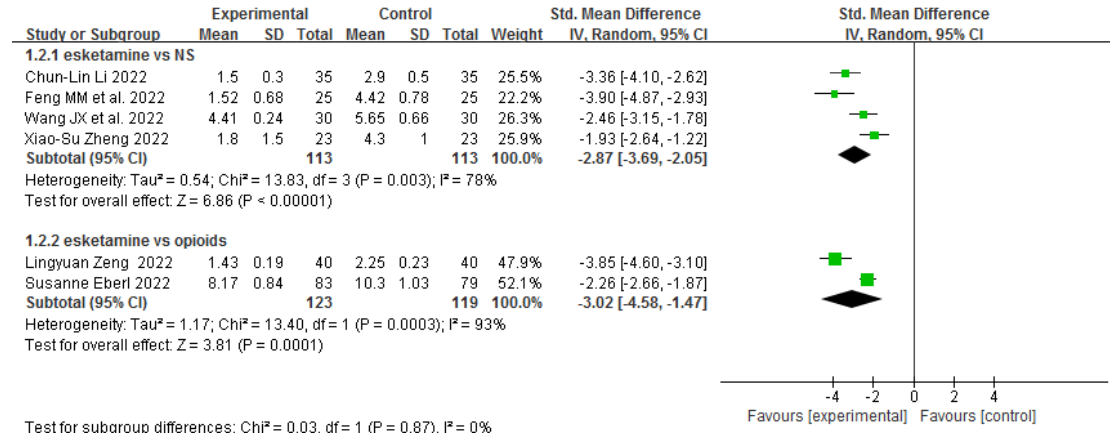

### (C) 0.7-1 mg/kg esketamine

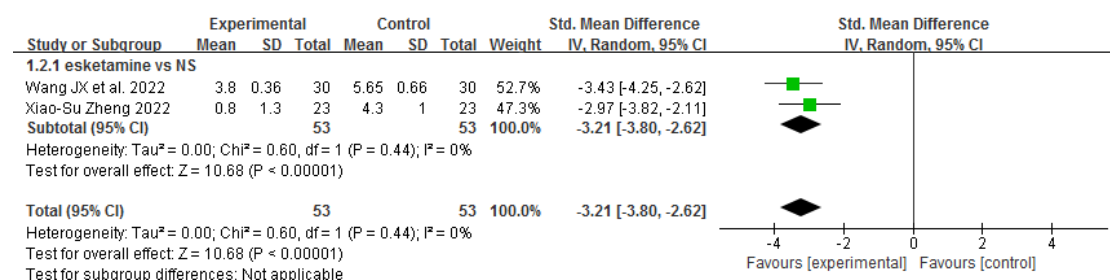

Supplement: Supplementary file 2 — Additional file 2: Figure S2. Forest plots of the propofol dose with different dosage of esketaime (mg/kg). [file 12871_2023_2167_MOESM2_ESM.pdf]

### (A) 0.1-0.15mg/kg esketamine

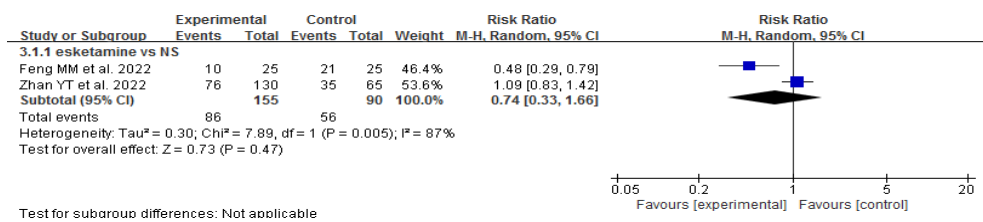

### (B) 0.2-0.3mg/kg esketamine

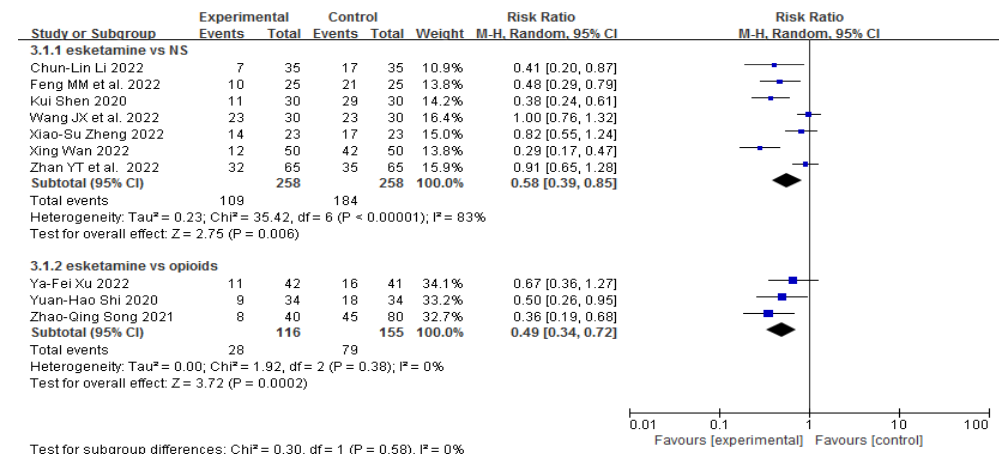

### (C) 0.4-0.5 mg/kg esketamine

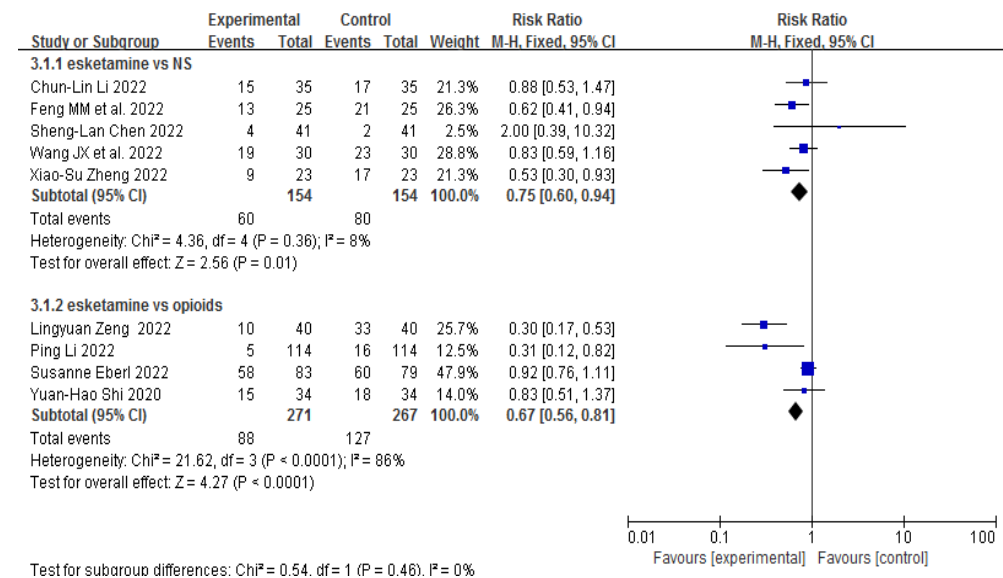

### (D) 0.7-1mg/kg esketamine

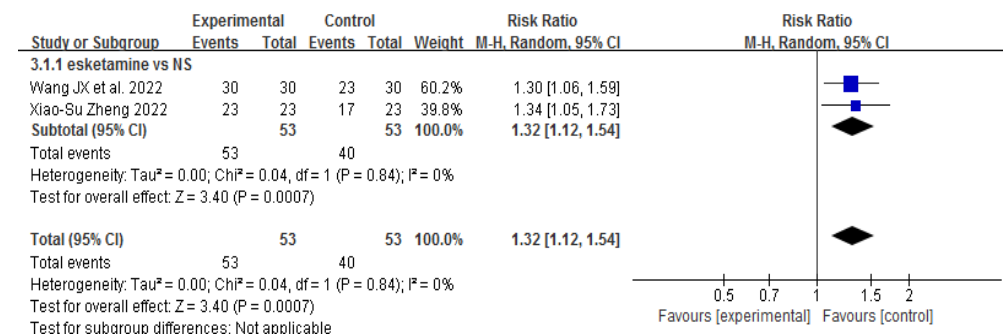

Supplement: Supplementary file 3 — Additional file 3: Figure S3. Forest plots of the complications with different dosage of esketaime (mg/kg). [file 12871_2023_2167_MOESM3_ESM.pdf]

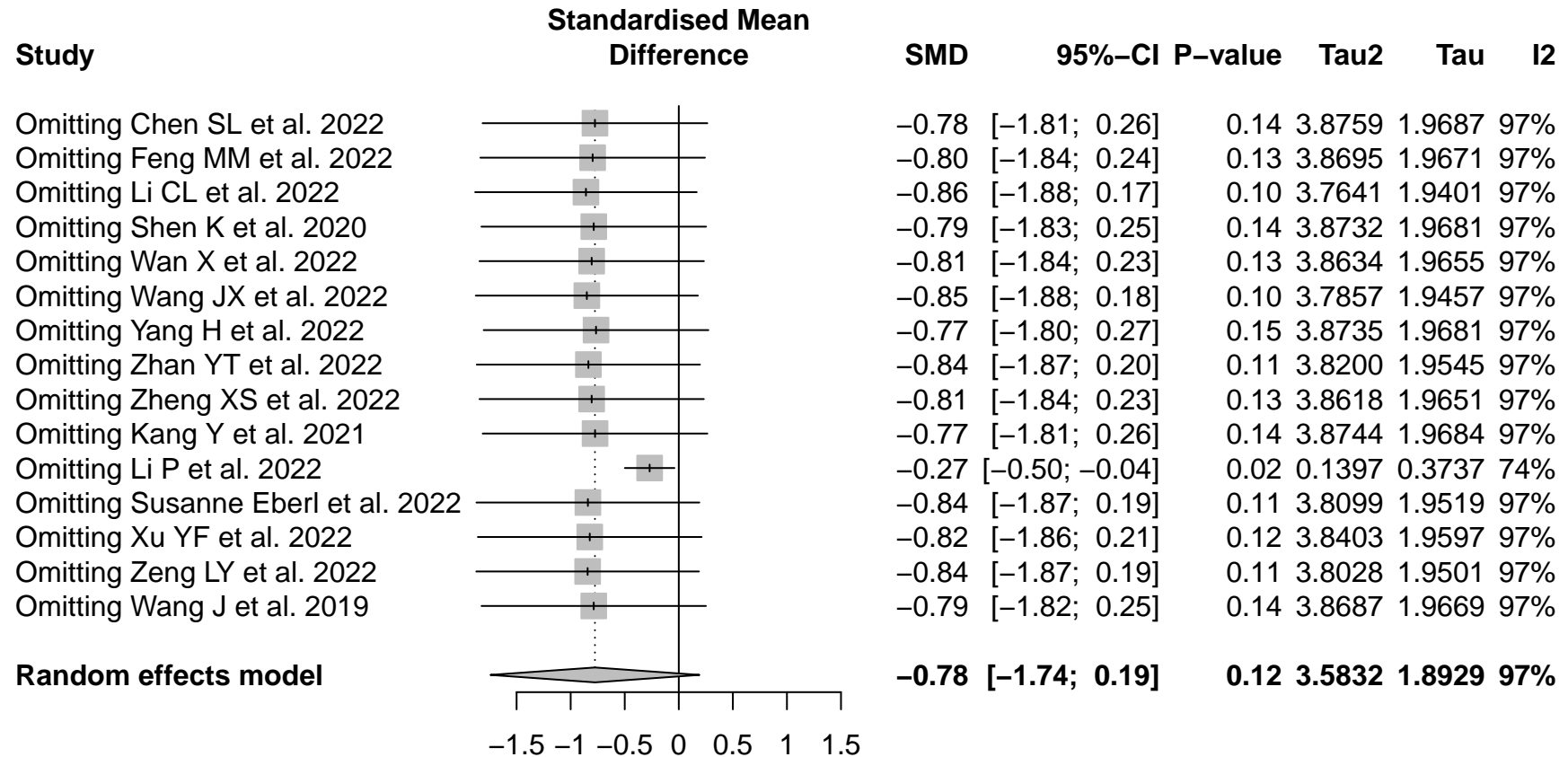

Supplement: Supplementary file 4 — Additional file 4: Figure S4. Forest plots of sensitivity of recovery time. [file 12871_2023_2167_MOESM4_ESM.pdf]

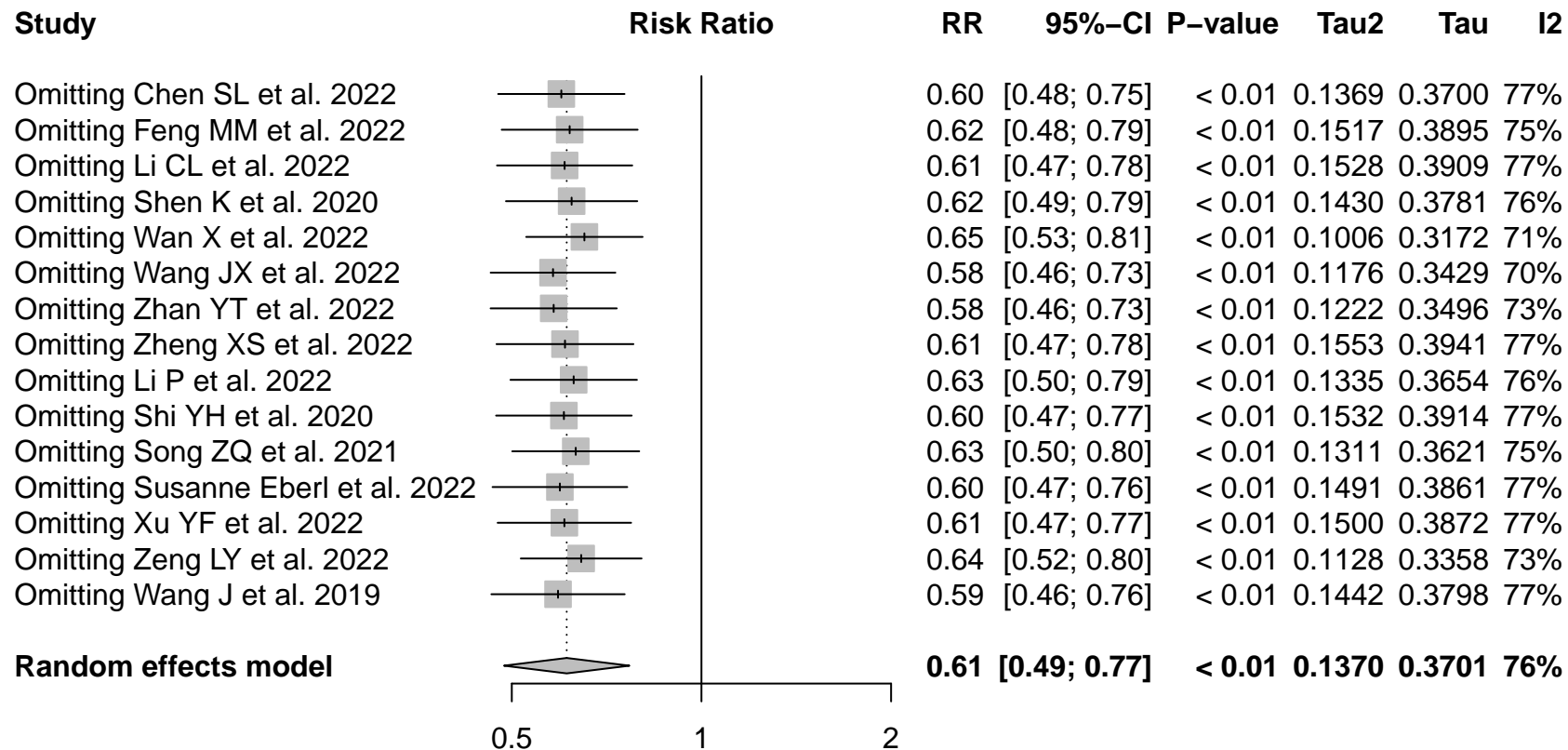

Supplement: Supplementary file 5 — Additional file 5: Figure S5. Forest plots of sensitivity of the adverse events. [file 12871_2023_2167_MOESM5_ESM.pdf]

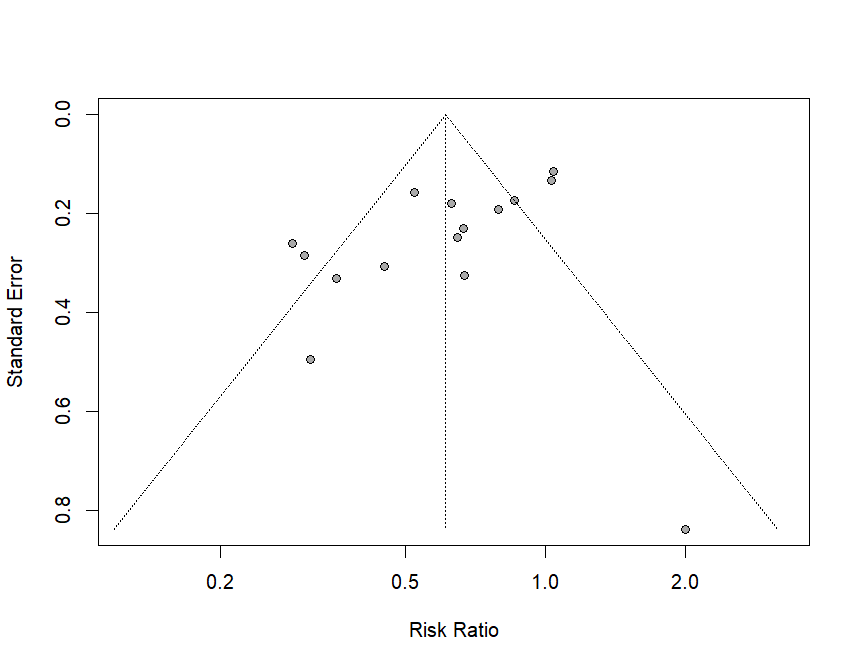

Supplement: Supplementary file 6 — Additional file 6: Figure S6. The funnel plot of adverse events. [file 12871_2023_2167_MOESM6_ESM.png]
