# Supplementary material for: Efficacy and safety of esketamine for sedation among patients undergoing gastrointestinal endoscopy: a systematic review and meta-analysis
Source: BMC Anesthesiol. 2023 Jun 13;23:204. doi: 10.1186/s12871-023-02167-0 (PMC10262466; doi:10.1186/s12871-023-02167-0)
Supplement: Supplementary file 8 — Additional file 8: Table S1. Subgroup analysis results of 0.5 mg/kg esketamine as the cutoff point. [file 12871_2023_2167_MOESM8_ESM.doc]

**TABLE**

**Table S1. Subgroup analysis results of esketamine dosage with 0.5 mg/kg as the cutoff point.**

| **Subgroup**  **Outcomes** | [**Control**](javascript:;) | **Number of studies** | **Results of heterogeneity test** | | **Meta analysis results** | |
| --- | --- | --- | --- | --- | --- | --- |
|  |  |  | **P value** | **I^2^** | **MD or RR (95%** CI**)** | **P value** |
| **(A)** **Recovery time** | | | | | | |
| 0.1-0.5 mg/kg esketamine | NS group | 9 | 0.003 | 66% | -0.91(-1.67,-0.15) | 0.02 |
|  | [opioid](javascript:;)s group | 5 | ＜0.001 | 88% | -1.11(-2.82,0.60) | 0.20 |
| 0.7-1 mg/kg esketamine | NS group | 2 | 0.008 | 86% | -0.12(-12.67,12.42) | 0.98 |
| **(B)** **Propofol dosage** | | | | | | |
| 0.1-0.5 mg/kg esketamine | NS group | 5 | ＜0.001 | 86% | -1.57（-1.84,-1.31） | ＜0.001 |
|  | [opioid](javascript:;)s group | 2 | 0.27 | 17% | -0.79 (-0.90, -0.68) | ＜0.001 |
| 0.7-1 mg/kg esketamine | NS group | 2 | 0.44 | 0% | -3.21(-3.80, -2.62) | ＜0.001 |
| **(C)** **Adverse events** | | | | | | |
| 0.1-0.5 mg/kg esketamine | NS group | 8 | ＜0.001 | 78% | 0.65(0.47,0.88) | 0.006 |
|  | [opioid](javascript:;)s group | 6 | 0.03 | 60% | 0.51(0.35,0.74) | 0.0003 |
| 0.7-1 mg/kg esketamine | NS group | 2 | 0.84 | 0% | 1.32(1.12, 1.54) | 0.0007 |
